# Supplementary figures and images for: Antipsychotic-induced weight gain and metabolic effects show diurnal dependence and are reversible with time restricted feeding
Source: Schizophrenia (Heidelb). 2022 Aug 30;8(1):70. doi: 10.1038/s41537-022-00276-2 (PMC9427943; doi:10.1038/s41537-022-00276-2)

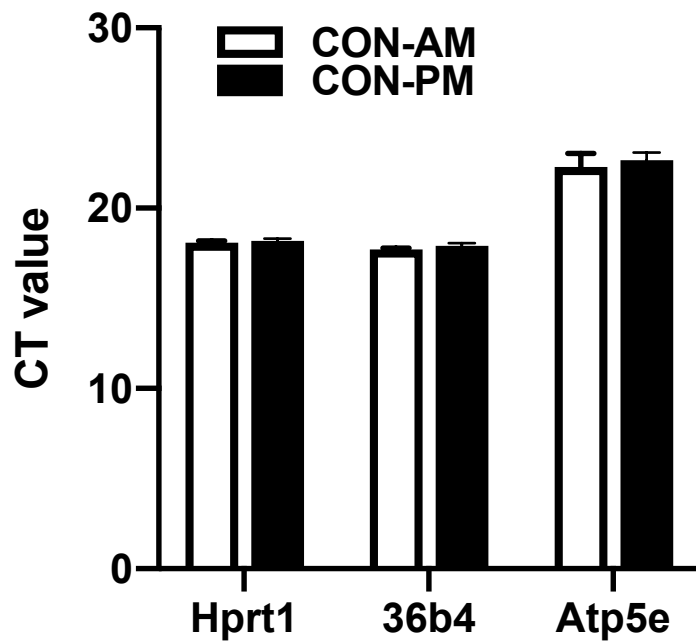

Supplement: Supplementary file 1 — supplemental figure 1 [file 41537_2022_276_MOESM1_ESM.pdf]
