# Supplementary material for: Antipsychotic-induced weight gain and metabolic effects show diurnal dependence and are reversible with time restricted feeding
Source: Schizophrenia (Heidelb). 2022 Aug 30;8(1):70. doi: 10.1038/s41537-022-00276-2 (PMC9427943; doi:10.1038/s41537-022-00276-2)
Supplement: Supplementary file 2 — supplementary materials [file 41537_2022_276_MOESM2_ESM.docx]

**Supplementary Materials**

**Supplemental figure 1. Relative expression of housekeeping genes used for qPCR analysis. Expression of** Hypoxanthine phosphoribosyltransferase 1 (Hprt1) for the hypothalamus (t=0.49, df=12.66, p=0.62), Acidic ribosomal phosphoprotein P0 (36B4) for the liver (t=1.21, df=10.36, p=0.25) and ATP synthase F1 subunit epsilon (Atp5e) for adipose tissues (t=0.40, df=11.39, p=0.69) are stable when comparing levels in tissues dissected at ‘AM’ and ‘PM’ timepoints. CON-AM n=7-8, CON-PM n=8. Data is expressed as mean ± SEM and analyzed using Student T-test. Significance was set at *p* < 0.05
